# Labor migration, the food supply chain and the COVID-19 syndemic: Germany, Netherlands, and the USA

**DOI:** 10.1093/eurpub/ckac129.405

**Published:** 2022-10-25

**Authors:** I Jungwirth, M Glassner

**Affiliations:** Society and Economics, Rhine-Waal University, Kleve, Germany

## Abstract

In Germany, the COVID-19 syndemic has brought questions regarding precarious labor migration in the food supply chain (FSC) to the forefront of the public debate. An analysis of responses to COVID-19-related challenges in agriculture and meat-processing pinpoints similarities and differences between the two industries, offering lessons for workers’ health and safety, and for the improvement of the FSC's systemic resilience. Agriculture and meat-processing were highlighted as “essential” during the COVID-19 syndemic in Germany. In agriculture, amidst lockdowns and border closures, special arrangements allowed permits for migrant workers to harvest seasonal and perishable produce, thus ensuring food supply and mitigating economic loss. Bilateral agreeements with countries neighbouring the EU were concluded to increase temporary labor migration for seasonal work; while COVID-19 specific occupational health standards were set. In meat-processing, which emerged as a hotbed for COVID-19 in Germany, the public debate problematized the contradictions between migrant workers’ essential contribution to the economy on the one hand, and their poor working and living conditions on the other hand. Here, the pandemic led to new legislation, prohibiting subcontracting in the meat processing industry and requiring higher standards for the housing of temporary migrant workers. While the long-term outcomes of these reforms remain to be seen, enforcement of health standards and other social rights will need attention. The German case, thus, underscores the importance of structural changes for ensuring social protection and dignified working conditions for labor migrants, which can, in turn, improve fairness, sustainability and systemic resilience of the FSC.

